# A high salt diet impairs the bladder epithelial barrier and activates the NLRP3 and NF‑κB signaling pathways to induce an overactive bladder *in* *vivo*

**DOI:** 10.3892/etm.2024.12651

**Published:** 2024-07-12

**Authors:** Jingwen Xue, Zhipeng Zhou, Zhangrui Zhu, Qi Sun, Yuexuan Zhu, Peng Wu

**Affiliations:** 1 Department of Urology, Nanfang Hospital, Southern Medical University, Guangzhou, Guangdong 510515, P.R. China; 2 Department of Urology, Jinshan Branch of Fujian Provincial Hospital, Fuzhou, Fujian 350004, P.R. China

**Keywords:** overactive bladder, high salt diet, bladder epithelial barrier, oxidative stress, nucleotide-binding domain leucine-rich-containing family pyrin domain-containing 3 signaling, NF-κB signaling

## Abstract

Overactive bladder (OAB) is a condition characterized by an urgency to urinate, which is associated with the urodynamic observation of detrusor overexcitation. Although the etiology of OAB is currently unclear, it has been suggested that in patients with OAB, disruption of bladder epithelial barrier integrity can disturb the normal contractile function of the detrusor. Additionally, dietary preferences have been suggested to influence the severity of OAB. Therefore, the aim of the present study was to investigate the effect of a high salt diet (HSD) on the development of OAB in a murine model. Mice were fed either a HSD or standard diet for 8 weeks, following which voiding characteristics and bladder barrier function were assessed. The present study demonstrated that a HSD in mice was associated with OAB-like symptoms such as increased urinary frequency and non-voiding bladder contractions. The HSD group demonstrated a thinner bladder mucus layer and decreased expression of bladder barrier markers, tight junction protein-1 and claudin-1, which may be potentially indicative of induced bladder damage. A HSD for 8 weeks in mice and a high salt treatment at the uroepithelium cellular (SV-HUC-1s) level resulted in increased uroepithelial oxidative stress and inflammatory cell infiltration, as indicated by increased expression levels of TNF-α and IL-1β, as well as activation of the nucleotide-binding domain leucine-rich-containing family pyrin domain-containing 3 (NLRP3) and NF-κB signaling pathways *in vivo* and *in vitro*. Therefore, the present study indicated that a HSD could be a potentially important risk factor for the development of OAB, as it may be associated with overactivation of contractile function of the bladder by impairing the integrity of the bladder epithelial barrier and activation of the NLRP3 and NF-κB signaling pathways. Remodeling of the bladder barrier and reduction of the inflammatory response may be potential targets for the treatment of OAB in the future.

## Introduction

Lower urinary tract symptoms (LUTS) refers to a group of symptoms related to lower urinary tract diseases that are characterized by increased urinary frequency, urinary urgency, nocturia and dysuria. LUTS can be categorized according to storage, voiding and postmicturition symptoms. Storage LUTS includes urinary urgency, urinary frequency, nocturi and urinary incontinence (1). Overactive bladder (OAB) is classed as one of the representative diseases of storage LUTS (2). The International Continence Society defines the symptoms of OAB as an urgent urination need, with or without urinary incontinence, and increased daytime and nocturnal urination, exclusion of organic bladder pathology is required prior to OAB diagnosis (3). OAB is reported to have a high prevalence of 20.8% in the Asia-Pacific region and a high recurrence rate of symptoms after remission. Existing available OAB treatment options include behavioral training, drug intervention, detrusor injection and electrical stimulation of sacral or tibial nerves, but the curative effect of these options is limited (4,5).

The impact of diet and lifestyle habits on the occurrence of LUTS has been reported in a previous study (6). A high salt intake is a characteristic element of the Western diet, however, it has been demonstrated that a high salt intake can lead to urinary frequency and nocturia in various animal models (7,8). Although epidemiologic studies have suggested that a high salt diet (HSD) is associated with the development of OAB, current *in vivo* and *in vitro* studies on the association of a HSD with development of OAB are limited (7,9).

Although the etiology and pathogenesis of OAB is currently unclear, it has been suggested that the bladder epithelium might be involved in neural signaling as a receptor for tension, chemical or temperature stimuli, termed the epithelial origin theory (10,11). Damage to the epithelium has been suggested to increase the excitability of local afferent nerves, which in turn leads to unstable contraction of the detrusor and the development of OAB (12). Studies have reported that decreased expression levels of cell junction-associated proteins increases the permeability of the bladder epithelial layer in cystitis-related OAB (13,14). Increased permeability has been shown to promote the diffusion of urinary solutes across the urinary epithelial barrier, which increases bladder afferent nerve sensitivity and facilitates voiding at low filling volumes (15). A HSD has been reported to increase the permeability of barriers such as the intestinal barrier or blood brain barrier to trigger colitis or cerebral apoptosis (16,17). The excessive sodium load that enters the circulatory system following a HSD must be eliminated through the urine, which keeps the bladder in a state of high sodium urinary load (8). However, it is currently unclear whether a high salt environment affects the bladder epithelium barrier to induce OAD symptoms.

NLRP3 detects pathogens and cell damage, activating the NLRP3 inflammasome, which in turn activates caspase-1, leading to cytokine activation and pyroptosis. The inflammasome includes NLRP3, caspase-1 and ASC, connecting NLRP3 and caspase-1(18). NF-κB regulates immune responses, inflammation, and cell survival, activation and differentiation. Dysregulated NF-κB activation is linked to inflammatory diseases, such as rheumatoid arthritis, inflammatory bowel disease, multiple sclerosis and asthma (19). Elevated sodium intake in mice with hypertension increased NLRP3 signaling activation and IL-1β secretion, while NF-κB signaling induced the transcriptional expression of NLRP3 (19-21). The link between OAB, NLRP3 and NF-κB involves inflammatory pathways (22). NLRP3, a key component of the inflammasome, is elevated in OAB, promoting the secretion of pro-inflammatory cytokines such as IL-1β. NF-κB, a transcription factor, regulates NLRP3 expression, exacerbating inflammation and contributing to bladder detrusor overactivity in OAB (23). In a Sprague Dawley rats animal model of neurogenic bladder, inhibition of NF-κB signaling attenuated uroepithelial cell pyroptosis, thereby serving an important role in bladder epithelial barrier homeostasis (24). Tight junction proteins, including Claudins, Occludins, and Zonula occludens proteins, seal the space between adjacent cells in the bladder epithelial barrier, preventing pathogen entry and collectively maintaining barrier integrity and functionality (25). Therefore, it could be suggested that HSD-induced OAB may be associated with the activation of NLRP3 and NF-κB signaling pathways and bladder epithelial damage.

TRPV4 was localized to the epithelial and muscular layers of the bladder where it has been reported to sense various stimuli. TRPV4 is suggested to increase the sensitivity of the detrusor and excitation of the voiding reflex, as reported in previous OAB models (26,27).

The present study aimed to investigate the effects of HSD on bladder barrier function and the development of OAB. In order to investigate the underlying mechanisms of HSD-induced OAB and bladder epithelium damage, *in vivo* experiments to examine the voiding characteristics and bladder epithelial integrity of a HSD murine model were performed, followed by *in vitro* investigations of the effect of high salt on bladder epithelium damage.

## Materials and methods

### Murine model

All animal experiments were conducted in accordance with the National Institute of Health guidelines and were approved by the Institutional Animal Ethical Care Committee of Southern Medical University (approval no. NFYY-2021-0572; Guangzhou, China). Pathogen-free male C57BL/6 mice (age, 6-8 weeks; weight, 20-24 g) were purchased from SPF Biotechnology Co., Ltd. All mice were housed in a temperature- and humidity-controlled environment (22±1˚C; 50±5%) with a 12 h light/dark cycle and free access to food and water. Currently available literature suggests 0.49% NaCl (w/v) in drinking water as the normal salt diet for mice (28). HSD mice (HSD group, n=6) were provided with drinking water containing 2% (w/v) NaCl (1.7 g NaCl/mouse/week) for 8 weeks, in accordance with a previously published HSD murine model (29). Control mice (CON group, n=6) received normal drinking water during the experiment. In addition, CON and HSD groups were both provided with 0.3% (w/w) salt concentration feed, which is the normal concentration of NaCl in mice feed, according to the manufacturer's guidelines (SPF Biotechnology Co., Ltd). Urine was collected every week for further examination. After 8 weeks, the necks of the animals were severed under complete anesthesia achieved through an intraperitoneal injection of 1.5% (w/v) sodium pentobarbital (40 mg/kg). Death was confirmed by cardiac arrest, a drop in body temperature and a lack of response to strong stimuli. The tissue harvest time was ~10-15 min/mouse. Tissue was snap frozen with a freezing temperature of -80˚C. After euthanasia, serum was collected and bladder tissue harvested in a sterile manner for further analysis. Blood collection was performed by open laparotomy through the abdominal aorta using a fine needle to collect ~0.5-0.8 ml of blood. Each day, mice were weighed at a regular time, the cage bedding was changed and their health assessed. If mice were found unable to feed or drink, did not respond to gentle stimulation or experienced body weight loss >20% compared with their starting weight, they were considered to be unsuitable for further experimentation and were to be euthanized by cervical dislocation under general anesthesia using the aforementioned method. However, no animal in this experiment reached these humane endpoints.

### SV40 virus transformed human uroepithelium cells (SV-HUC-1s)

Human SV-HUC-1s (cat. no. CRL-9520; American Type Culture Collection) were cultured in Ham's F-12K medium (Gibco; Thermo Fisher Scientific, Inc.) supplemented with 10% fetal bovine serum (Gibco; Thermo Fisher Scientific, Inc.) and 0.5% penicillin-streptomycin (Gibco; Thermo Fisher Scientific, Inc.) under standard cell culture conditions (temperature, 37˚C; CO_2_, 5%). SV-HUC-1s were seeded into 6-well plates at a density of 6x10^4^ cells/well (n=6 well/treatment group) in culture media for 24 h for use in the reactive oxygen species (ROS), malondialdehyde (MDA) and myeloperoxidase (MPO) assays and reverse-transcriptase quantitative (RT-q) PCR. The concentration of NaCl in Ham's F-12K medium was 150 mM. During the logarithmic growth period, additional NaCl was added to the HSD group to reach a concentration of 190 mM for 24 h (21). CON cells were simultaneously maintained in standard Ham's F-12K media (150 mM) for 24 h.

### Urinary frequency measurement

Urgency is a subjective sensation that cannot intuitively be assessed in mice. Measurement of non-urinary contractions in urodynamic experiments was utilized as an indicator of urgency, which previous studies have reported to be an objective method of measuring a response to the sensation of urgency (30-32). Urinary frequency was measured as previously described (33). Briefly, individual mice were placed in a metabolic cage and abstained from water for 4 h. A sheet of copper sulfate paper was placed at the bottom of each cage. Urinary frequency was determined by the number of voiding spots on the filter paper. Overlapping urine spots with defined edges were considered to be separate urinations. Urination frequency was recorded for 3 consecutive days and the average of the three days' values were calculated.

### Cystometry

Mice were anesthetized using 3.0-4.0% isoflurane by inhalation for induction of anesthesia and anesthesia maintained using 1.0-1.5% isoflurane while the mouse was placed in the supine position. The lower abdomen was excised to fully to expose the bladder. An intravenous needle (0.6x15 mm) was gently inserted into the bladder and secured and a 1.0 ml syringe was used to withdraw residual urine. The BL-420N BioSignal Acquisition System (Chengdu Techman software Co., Ltd.) was used as a pressure measurement device. Sterile saline at 0.9% (w/v) was continuously pumped into the bladder at a flow rate of 1 ml/h. When a consistent and stable, the non-interfering cluttered waveform of micturition was established, digital intravesical pressure signals were continuously recorded for 30 min or for at least five void cycles. During the bladder filling period, it was observed if urine flowed out of the external urethra of the mice. When the bladder of mice contracts, there is a noticeable increase in pressure on the urodynamic chart, and urination occurring simultaneously is termed micturition contraction. When the bladder of mice contracts without urination, it is defined as non-micturition contraction (34). When the bladder contracts and urination occurs, the pressure recorded on the urodynamic chart represents the peak pressure and the maximum bladder capacity was calculated (maximum bladder capacity=perfusion time x perfusion rate).

### Gene expression analysis

Total RNA was extracted from whole bladder tissue or SV-HUC-1s using either the Animal Total RNA Isolation Kit or the Cell Total RNA Isolation Kit, respectively (cat. no. RE-03011/03014; Foregene Co., Ltd.) according to the manufacturer's instructions. A reverse transcriptase (RT) enzyme, HiScript III RT SuperMix for qPCR + gDNA wiper (Vazyme Biotech Co., Ltd.; cat. no. R323-01), was used to obtain cDNA from total RNA (temperature and duration: 50˚C for 15 min; 85˚C for 5 sec). The RT-qPCR reactions (initial denaturation: 95˚C for 30 sec; 40 cycles of amplification at 95˚C for 10 sec and 60˚C for 30 sec; followed by melting curve analysis at 95˚C for 15 sec, 60˚C for 1 min and 95˚C for 15 sec) were performed using the ChamQ SYBR qPCR Master Mix (Vazyme Biotech Co., Ltd.), using the LightCycler480 (Roche Diagnostics). GAPDH was used as the internal reference gene. Relative quantification of target genes were calculated using the 2^-∆∆Cq^ method (35). All sequences of primers utilized are listed in Table I.

### Histological staining and analysis

The bladder tissue samples of mice were collected, sliced horizontally and fixed in 4% paraformaldehyde for 3 days at room temperature. The samples were embedded in paraffin, sectioned at 4-µm thick and stained with hematoxylin and eosin (H&E; hematoxylin for 4 min and eosin for 20 sec) at room temperature. The method of histologic scoring was as previously described (36). Briefly, the histologic score was determined by the degree of bladder edema, inflammatory cell infiltration, bleeding and ulcer formation and depth of mucosal injury (absent, 0; mild, 1; moderate, 2; severe, 3). Measurements of the thickness of mucous layer and lamina propria was obtained from three randomly selected regions of a tissue section from each sample and the mean thickness was calculated. Immunohistochemistry was performed as follows: Antigen retrieval was performed with 0.01 M, pH 6.0 sodium citrate solution for 10 min in a microwave at 98˚C and then allowed to cool down at room temperature. Endogenous peroxidase activity was blocked with 0.3% hydrogen peroxide at room temperature for 10 min, followed by incubation with 5% goat serum (cat. no. BL210A; Biosharp Life Sciences) for 30 min at room temperature. The samples were then incubated at 4˚C overnight with the following primary antibodies: Rabbit anti-TJP-1 antibody (1:250; cat. no. ab276131; Abcam), rabbit anti-CLAUDIN-1 antibody (1:200; cat. no. YT0942; ImmunoWay Biotechnology Company), rabbit anti-TRPV4 antibody (1:200; cat. no. YT5833; ImmunoWay Biotechnology Company), rabbit anti-CASP1 antibody (1:200; cat. no. YP0749; ImmunoWay Biotechnology Company) and rabbit anti-NF-κB antibody (1:200; cat. no. YM8001; ImmunoWay Biotechnology Company). Subsequently, the samples were incubated with horseradish peroxidase-conjugated goat anti-rabbit secondary antibody at 37˚C for 30 min (1:200; cat. no. LF102; Shanghai Epizyme Biotech Co., Ltd.). DAB solution (cat. no. BL732A; Biosharp Life Sciences) was added and the samples were incubated 3 min. Counterstain sections were immersed in hematoxylin and 0.1% HCl- ethanol for 1-10 sec, and washed with distilled water. Samples were dehydrated through 95% ethanol for 1 min, 100% ethanol for 2 min, xylene for 2 min, and then immersed with the coverslip with mounting medium. All images were scanned by a NanoZoomer Digital slide scanner and captured with an NDP View2 Plus Image viewing software (version U12388-01; Hamamatsu Photonics K.K.). Quantification of the average optical density was performed using the Image-Pro Plus software (version 6.0; Media Cybernetics, Inc.) in three randomly chosen fields of view, at x10 magnification, from each sample.

### Biochemical and oxidative stress marker analysis

Serum and urine Na^+^, K^+^, Ca^2+^ and Cl^-^ concentrations were determined using an automatic biomedical analyzer (Roche Diagnostics). Mice urinary proteins were detected using an ELISA kit, according to the manufacturer's protocol (cat. no. MM-44286M2; Shanghai MEIMIAN Biotechnology, Co., Ltd.). MDA levels were detected using an ELISA kit, according to the manufacturer's protocol (cat. no. E-EL-0060; Wuhan Elabscience Biotechnology Co., Ltd.). MPO levels were detected using the MPO Activity Assay kit, according to the manufacturer's protocol (cat. no. E-BC-K074-M; Wuhan Elabscience Biotechnology Co., Ltd.). MPO and MDA levels were evaluated using the homogenate of the cell culture, whereby cells were digested for 2 mins, centrifuged at 20˚C and 200 x g for 3 min and resuspended for use).

### Intracellular ROS generation determination

Intracellular ROS was detected using a ROS assay kit (cat. no. S0033S; Beyotime Institute of Biotechnology). Briefly, SV-HUC-1 cells were cultured in 6-well plates and incubated with fluorescent 2',7'-dichlorofluorescein diacetate (Beyotime Institute of Biotechnology) for 20 min at 37˚C. The fluorescent ROS signals were detected in darkness and images captured using an excitation wavelength of 488 nm and an emission wavelength of 525 nm using an inverted fluorescence microscope (ECLIPSE Ti2; Nikon Corporation). The ROS levels of each group were calculated using the Image-Pro Plus software. The average fluorescence intensity of each sample was calculated in relation to a reference value obtained from the average fluorescence intensity of the CON group.

### Statistical analysis

Data were presented as mean ± SD or median with interquartile range and were analyzed using GraphPad Prism software (version 8.0; Dotmatics). Statistical analyses were performed using a two-tailed unpaired Student's t-test or the Mann-Whitney U test for non-normal distributions, as indicated in the figure legends. P<0.05 was considered to indicate a statistically significant difference. Correlation between variables was calculated using Spearman correlation analysis. Consideration was only given to correlation values ρ>0.6 and P<0.05. Correlation network graphs were plotted using Wekemo Bioincloud software (www.bioincloud.tech).

## Results

### A HSD in mice altered micturition characteristics

**Figure 1:**
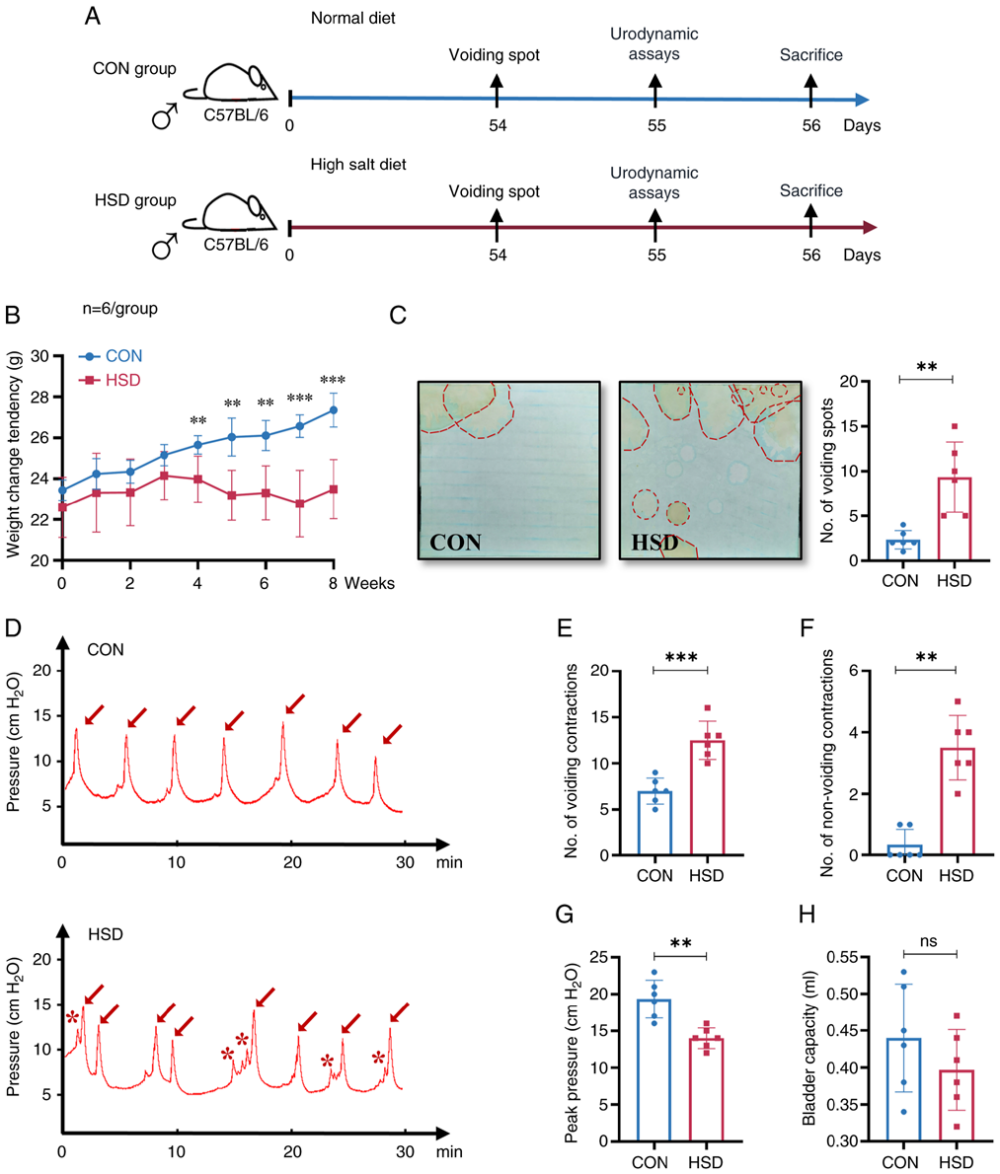
HSD altered micturition characteristics in mice. (A) Flowchart depicting the experiment timeline of mice either in HSD group or CON group for 8 weeks. (B) Weight measurements of HSD and CON mice over 8 weeks. (C) Representative images of continuous voiding spots during a 4 h period and quantification of the urination frequency. The red dashed lines marked the location of the voiding spots. (D) Representative traces of *in vivo* cystometry urodynamic assay during a 30 min continuous monitoring performed on the CON group and HSD group. Arrowheads indicate micturition events during the experiment and asterisks indicate non-voiding contractions of the bladder. Cystometric parameters between the CON group and HSD group were compared: (E) Number of voiding contractions, (F) number of non-voiding contractions, (G) maximum bladder pressure and (H) maximum bladder capacity. Data are presented as mean ± SD (n=6). P-values were calculated using a two-tailed unpaired Student's t-test; ^***^P<0.001 and ^**^P<0.01. HSD, high salt diet; CON, control; ns, non-significant.

In the present study, mice were placed on either a standard salt intake diet or a HSD for 8 weeks, following which changes in bladder and voiding behavior were assessed (Fig. 1A). An 8 week HSD significantly decreased the rate of weight gain of the HSD group compared with CON group (Fig. 1B; Table II). There were no significant differences in serum Na^+^ and Cl^-^ concentrations in the HSD group compared with the CON group (Table II). The concentration of Na^+^ and Cl^-^ in the urine of the HSD group was 4-fold higher compared with the CON group. Furthermore, a HSD did not significantly change the urine concentrations of K^+^ and Ca^2+^, however, urine protein expression levels were significantly increased in the HSD group compared with the CON group. Measurement of the number of voiding spots indicated that the HSD group urinated significantly more often compared with the CON group and the location of voiding tended to be in the middle of the cage in the HSD group compared with the CON group (Fig. 1C). Previous behavioral studies have reported that mice are more inclined to urinate in corners (37). In the present study, the HSD group tended to urinate more in the middle of the cage, which suggested that they were potentially more likely to experience frequent micturition compared with the CON group. Furthermore, the cystometry curve demonstrated that the number of voiding contractions and the number of non-voiding contractions were significantly increased in the HSD group compared with the CON group (Fig. 1D-F). A significant decrease in the maximum bladder pressure in the HSD group compared with the CON group was observed (Fig. 1G). There was no significant difference in bladder capacity of the HSD group compared with the CON group (Fig. 1H). The aforementioned results indicated that an 8 week HSD altered micturition characteristics and resulted in OAB-like symptoms in mice.

### A HSD in mice promoted an inflammatory response and impaired barrier integrity of the bladder

**Figure 2:**
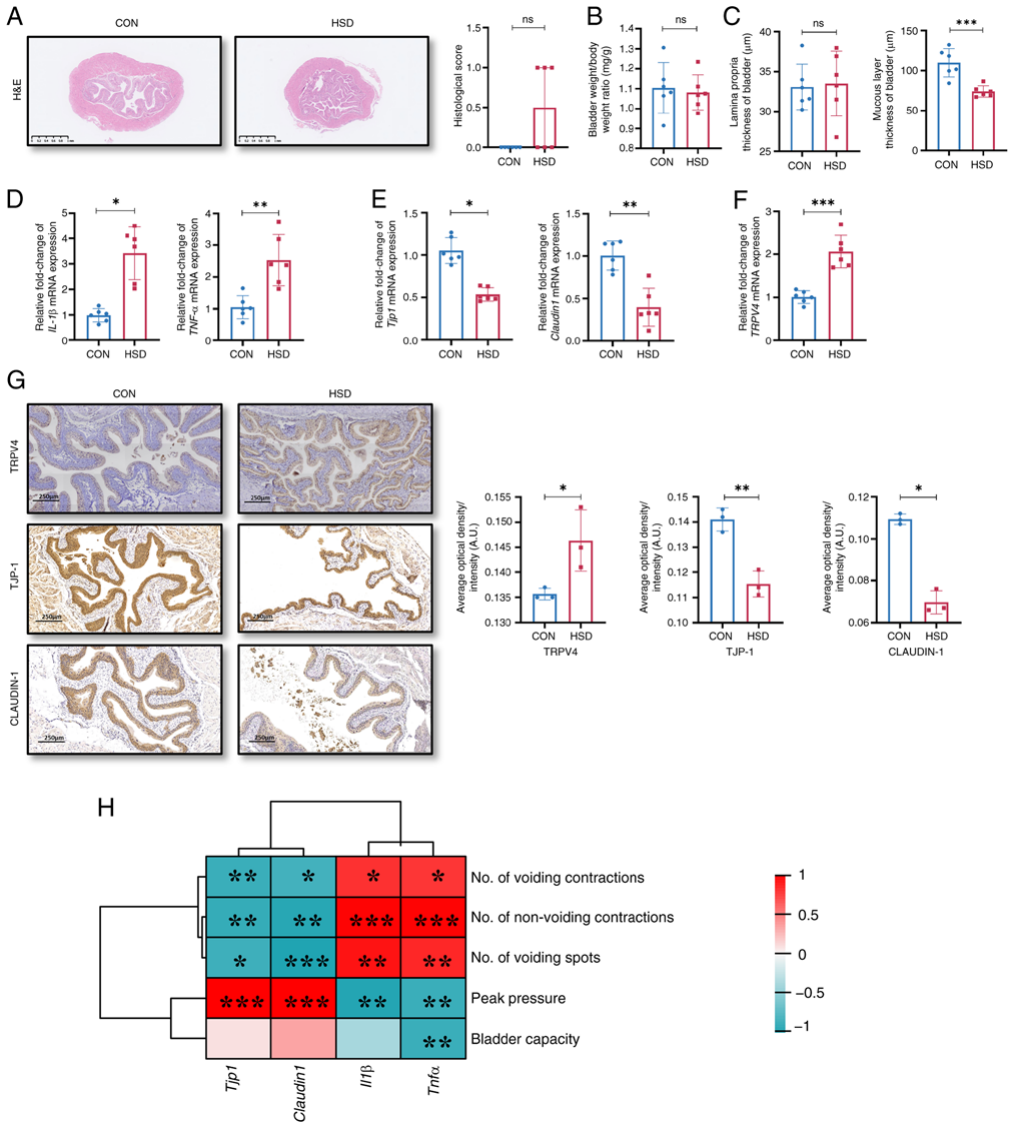
A HSD *in vivo* impaired barrier function of bladder. (A) H&E staining and histological score of bladder tissues (scale bar, 1 mm). Data were presented as the median with interquartile range. (B) Bladder weight/body weight ratio. (C) Thickness of lamina propria and mucosal layer of the bladder. Relative mRNA expression levels of (D) inflammatory response markers, IL-1β and TNF-α, (E) tight junction proteins, TJP-1 and Claudin-1 and (F) TRPV4. (G) Representative images of histological staining and quantification of protein expression of TRPV4, TJP-1 and CLAUDIN-1 in bladder tissues sections from CON and HSD mice. Scale bar, 250 µm (n=3). (H) Correlation analysis between the mRNA expression levels of tight junction proteins and inflammation factors in the bladder and urination characteristics in CON and HSD mice. Data are presented as mean ± SD (n=6). P-values were calculated using a two-tailed unpaired Student's t test; ^***^P<0.001, ^**^P<0.01 and ^*^P<0.05. HSD, high salt diet; CON, control; TJP-1, tight junction protein 1; TRPV4, transient receptor potential vanilloid 4; ns, non-significant; A.U., arbitrary units.

Histological scores of the bladder and the bladder weight/body weight ratio showed no significant differences between the HSD and CON groups (Fig. 2A and B), which was consistent with the clinicopathologic features of patients with OAB (3). However, the histological scores of the bladder in the HSD group were markedly higher compared with the CON group; therefore, the thickness of the mucosal layer and lamina propria of the bladder were separately measured. No significant difference in the thickness of the lamina propria was observed; however, the bladder mucosa layer of the HSD group was significantly thinner compared with that of the CON group (Fig. 2C). The mRNA expression levels of the pro-inflammatory factors IL-1β and TNF-α in the bladder were significantly higher in the HSD group compared with the CON group (Fig. 2D). The mRNA and protein expression levels of TJP-1 and CLAUDIN-1 in the bladder epithelium were significantly lower, while the levels of TRPV4 were significantly increased in the HSD group compared with the CON group (Fig. 2E-G). These results suggested that a HSD in mice impaired the integrity of the bladder epithelial barrier and potentially caused an inflammatory response.

### A HSD increased uroepithelial oxidative stress and affected NLRP3 and NF-κB signaling pathways

The mRNA and protein expression levels of tight junction proteins, TJP-1 and CLAUDIN-1 in the bladder was significantly negatively correlated with the number of voiding contractions, non-voiding contractions and voiding spots, and was significantly positively correlated with peak pressure (Fig. 2H). However, the mRNA expression levels of inflammatory factors, IL-1β and TNF-α, in the bladder were significantly positively correlated with OAB-like voiding behaviors, moreover, TNF-α level was significantly negatively correlated with bladder capacity.

**Figure 3:**
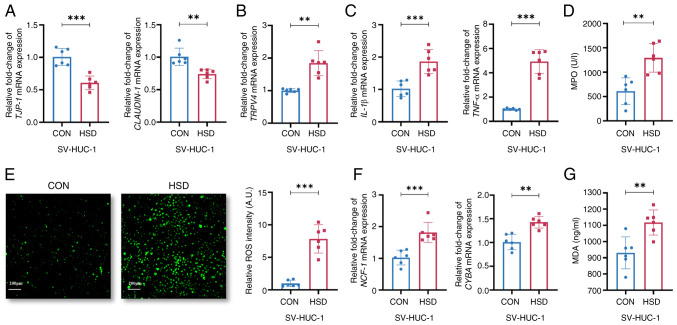
A HSD increased uroepithelial oxidative stress in SV-HUC-1 cells. Relative mRNA expression levels of (A) TJP-1 and CLAUDIN-1, (B) TRPV4 and (C) IL-1β and TNF-α in HSD-treated and CON cells. (D) Relative MPO expression levels in CON and HSD groups. (E) Representative images and quantification of intracellular ROS levels (scale bar, 200 µm). (F) Relative mRNA expression levels of NCF-1 and CYBA. (G) Relative MDA expression levels in CON and HSD groups. Data are presented as mean ± SD (n=6). P-values were calculated using a two-tailed unpaired Student's t-test; ^***^P<0.001 and ^**^P<0.01. HSD, high salt diet; CON, control; SV-HUC-1, SV40 virus transformed human uroepithelium cells; TJP-1, tight junction protein 1; TRPV4, transient receptor potential vanilloid 4; MPO, myeloperoxidase; ROS, reactive oxygen species; NCF-1, neutrophil cytosolic factor 1; CYBA, cytochrome B-245 alpha chain; MDA, malondialdehyde.

Previous studies have reported that oxidative stress and the NLRP3 and NF-κB signaling pathways are important for the maintenance of bladder epithelial cell homeostasis both in animals and *in vitro* models of bladder cancer and interstitial cystitis (24,38,39). However, to the best of our knowledge, whether the aforementioned pathways are altered in a HSD-triggered OAB model has not been reported to date. Therefore the present study undertook a series of *in vitro* experiments to examine the role of oxidative stress, NLRP3 and the NF-κB signaling pathways in HSD-induced OAB. The mRNA expression levels of TJP-1 and CLAUDIN-1 were significantly reduced, whereas the TRPV4 expression level was significantly increased in the HSD group compared with the CON group (Fig. 3A and B), which were consistent with the aforementioned results of the *in vivo* HSD model in the present study. The mRNA expression levels of IL-1β and TNF-α, as well as the relative expression levels of MPO, were significantly increased in the HSD group compared with the CON group (Fig. 3C and D). The fluorescence intensity of ROS was significantly higher in the HSD group compared with the CON group (Fig. 3E). The mRNA expression levels of neutrophil cytosolic factor 1 and cytochrome B-245 alpha chain, which are structural protein components of NADPH oxidase, were significantly higher in the HSD group compared with the CON group (Fig. 3F). MDA, an indicator of lipid peroxidation, was significantly increased in the HSD group compared with the CON group (Fig. 3G). This suggested a that high salt culture environment led to a higher level of oxidative stress *in vitro*.

**Figure 4:**
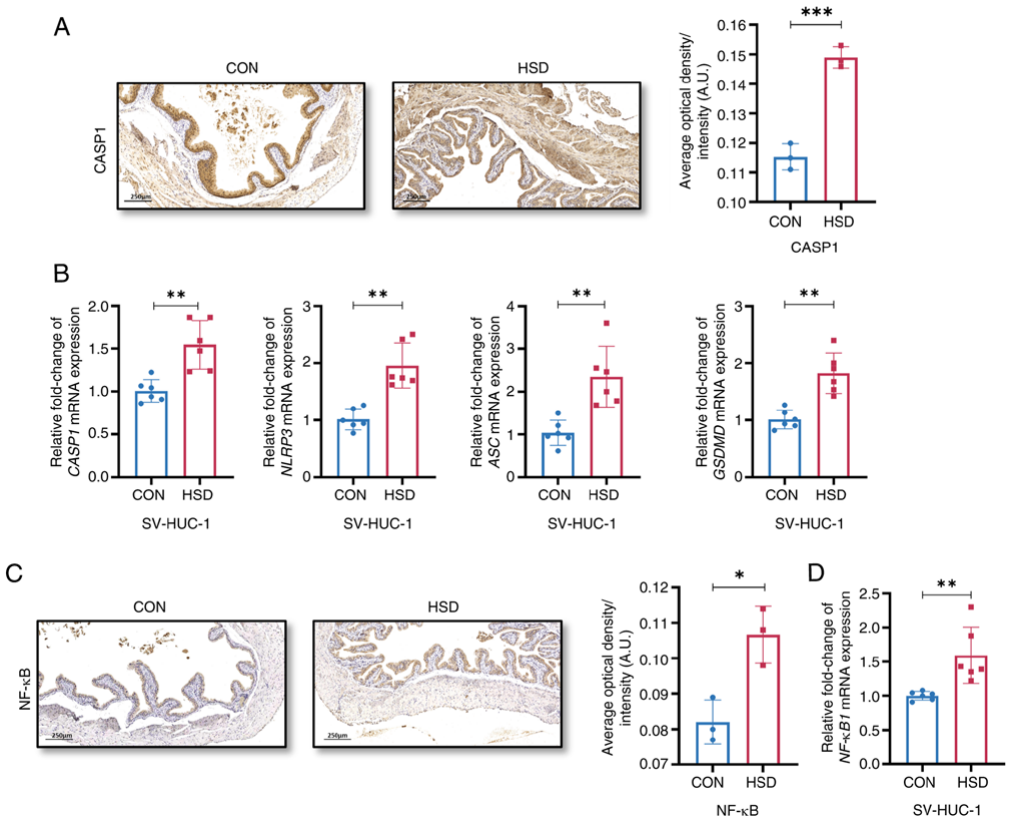
A HSD activated NLRP3 and NF-κB signaling pathways. (A) Representative histological images and quantification of NLRP3 signaling component, CASP-1 expression *in vivo* (n=3). (B) Relative mRNA expression levels of CASP1, NLRP3, ASC and GSDMD in CON and HSD treated SV-HUC-1 cells (n=6). (C) Representative histological images and quantification of NF-κB expression *in vivo* (n=3). (D) Relative mRNA expression levels of NF-κB1 in CON and HSD treated SV-HUC-1 cells (n=6). Data are presented as the mean ± SD. P-values were calculated using a two-tailed unpaired Student's t-test; ^***^P<0.001, ^**^P<0.01 and ^*^P<0.05. HSD, high salt diet; CON, control; SV-HUC-1, SV40 virus transformed human uroepithelium cells; CASP1, caspase-1; ASC, apoptosis-associated speck-like protein containing a caspase recruitment domain; NLRP3, nucleotide-binding oligomerization domain, leucine rich repeat and pyrin domain containing 3; GSDMD, gasdermin D.

The protein expression level of CASP1 and the mRNA expression levels of CASP1, NLRP3, apoptosis-associated speck-like protein containing a caspase recruitment domain and gasdermin D were significantly increased in the HSD group compared with the CON group (Fig. 4A and B). The protein and mRNA expression levels of NF-κB and NF-κB1, respectively, were significantly increased in the HSD group compared with the CON group (Fig. 4C and D), which suggested that high salt treatment affected NLRP3 and NF-κB signaling pathways in the urinary epithelium *in vitro* and *in vivo*.

## Discussion

The present study demonstrated that an 8 week HSD *in vivo* mouse model induced the increased expression of inflammatory state markers in the bladder, reduced gene and protein expression levels of bladder epithelial tight junction proteins, TJP-1 and CLAUDIN-1, and increased both protein and mRNA expression levels of TRPV4, which were associated with the development of OAB-like symptoms. In addition, the present study utilized a human cell line and demonstrated that high salt intervention increased oxidative stress level and elevated gene and protein expression levels of the NF-κB and NLRP3 pathway *in vitro*. To the best of our knowledge, the present study is the first to report that a HSD-induced inflammatory response of the bladder and OAB-like symptoms *in vivo* may be associated with activation of oxidative stress, NLRP3 and NF-κB signaling.

The ability of the body to maintain urine within the healthy concentration range relies upon the selective regulation of molecules according to molecular size and charge by tight junctions strands between bladder epithelial cells (40). The function of the bladder epithelial barrier is to monitor the mechanical and chemical environment of the bladder and transmit environmental alternations to the underlying tissues, such as afferent nerve fibers and smooth muscle (41). In the present study, changes in the ion concentration and albumin of urine in the HSD group indicated potential impairment of the bladder epithelial barrier function. Previous studies have shown that defects of proteins expression in uroepithelial tight junction formation, in particular tight junction proteins CLAUDIN-1 and occludin, led to sensory afferent nerve activation and caused pelvic pain, urinary frequency and urinary urgency (42,43). The present study demonstrated that gene and protein expression levels of tight junction proteins TJP-1 and CLAUDIN-1 in the bladder epithelium of mice were significantly reduced in an *in vivo* model of HSD and were significantly negatively correlated with OAB-like symptoms. The aforementioned results suggested that a decrease in bladder tight junction protein expression level may have been a potential cause of bladder epithelial barrier impairment and the occurrence of OAB-like symptoms in the HSD group. The TRPV4 channel senses chemical stimuli in the bladder and the increased expression of TRPV4 has been previously reported to induce sensitization of bladder afferent nerves and the onset of the voiding reflex. TRPV4 has been reported to directly modulate the contractility of the detrusor, which was associated with the development of OAB (26,44). TRPV4 is also associated with urothelial barrier function and a previous study has demonstrated that the integrity of the bladder epithelium was disrupted in TRPV4^-/-^ mice (26). It could be suggested that increased TRPV4 expression levels due to the *in vivo* model of HSD that may have potentially contributed to a positive feedback regulation of epithelial barrier damage in the present study. In summary, the present study demonstrated that an 8 week HSD *in vivo* could lead to upregulation of markers of bladder barrier damage and OAB-like symptoms.

Previous studies have suggested that inflammatory responses induced by a HSD are associated with the activation of oxidative stress and NLRP3 signaling markers. NF-κB signaling is suggested to increase NLRP3 transcription, which together act synergistically to induce a pro-inflammatory response (19,20). In the present study, the activation of NLRP3 and NF-κB signal pathway were obtained in an *in vivo* model of HSD-induced bladder dysfunction. Bladder C fibers refer to a specific type of nerve fibers, which play an important role in controlling the process of urination, helping the brain perceive the status of the bladder and regulate the timing of urination. In addition, NLRP3 activation has been reported to increase C-fiber populations in the bladder and lead to OAB-like symptoms in diabetic mice (45). It was also reported that NLRP3 activation impaired the integrity of the urothelium barrier, and the down-regulation of tight junction protein expression levels was observed in the overactive stage in NLRP3^-/-^ diabetic mice (46). A previous study further showed that activation of the NF-κB signaling pathway inhibited the transcription of caveolins and was associated with smooth muscle hypertrophy in the bladder, which interfered with normal bladder contraction in humans and mice (47). To the best of our knowledge, the present study was the first to report that OAB-like symptoms induced by HSD were associated with alterations in the NLRP3 and NF-κB signaling pathways, as well as inducing the loss of urothelium tight junction proteins.

The present study had a number of limitations. The results drawn from the expression levels of tight junction proteins cannot be entirely conclusive in terms of altered bladder barrier function, although the present data provided some representative indication of function. Due to limitations in the experimental conditions of the present study, functional experiments, such as measurement of transepithelial resistance, could not be performed. Additionally, the TRPV ion channel family has many members, such as TRPV1, that are also expressed in the bladder epithelium and muscularis propria (48). In the present study, the effects of a HSD regime on alternate TRPV receptors, other than TRPV4, was not observed. Therefore, the specific mechanisms underlying HSD-induced bladder barrier damage warrants further investigation. Based on the research presented in the current study, an appropriate dietary salt intake for humans could not be recommended, as this requires further clinical studies to be performed in the future.

In conclusion, the present study showed that an 8 week HSD in mice could induce OAB-like symptoms, which potentially may have been associated with disruption of the bladder epithelial barrier integrity, as well as increased TRPV4 expression levels. It was demonstrated that high-salt treatment *in vitro* increased uroepithelial oxidative stress and activation of the NLRP3 and NF-κB signaling pathways. Results from the present study highlighted the importance of the structural integrity of the bladder barrier on the development of OAB and these results could potentially suggest new avenues of future treatment for patients with OAB.

## Figures and Tables

**Table I:** Primer sequences for reverse transcription-quantitative PCR.

Gene		Sequence (5'-3')
Mouse	Claudin1	F: GCCATCTACGAGGGACTGTG
		R: CCCCAGCAGGATGCCAATTA
	TJP1	F: AGAGACAAGATGTCCGCCAG
		R: TGCAATTCCAAATCCAAACC
	IL-1β	F: CAGGCAGGCAGTATCACTCA
		R: TGCAATTCCAAATCCAAACC
	TNF-α	F: AGGGTCTGGGCCATAGAACT
		R: CCACCACGCTCTTCTGTCTAC
	TRPV4	F: CGGACCACAGTGGACTACCT
		R: GAGACAACCACCAGCACAGA
	GAPDH	F: AGGTCGGTGTGAACGGATTTG
		R: TGTAGACCATGTAGTTGAGG
		TCA
Human	TNF-α	F: TCCTTCAGACACCCTCAACC
		R: AGGCCCCAGTTTGAATTCTT
	IL-1β	F: GGGCCTCAAGGAAAAGAATC
		R: TTCTGCTTGAGAGGTGCTGA
	TJP-1	F: TGAGGCAGCTCACATAATGC
		R: GGTCTCTGCTGGCTTGTTTC
	CLAUDIN-1	F: CCGTTGGCATGAAGTGTATG
		R: CCAGTGAAGAGAGCCTGACC
	TRPV4	F: GCGAGGTCATTACGCTCTTC
		R: TAGAGGGCTGCTGAGACGAT
	NCF-1	F: AGTCCTGACGAGACGGAAGA
		R: TACATGGACGGGAAGTAGCC
	CYBA	F: CGCTTCACCCAGTGGTACTT
		R: GAGAGCAGGAGATGCAGGAC
	CASP1	F: GCTTTCTGCTCTTCCACACC
		R: CATCTGGCTGCTCAAATGAA
	ASC	F: TGACGGATGAGCAGTACCAG
		R: TCCTCCACCAGGTAGGACTG
	NLRP3	F: CTTCTCTGATGAGGCCCAAG
		R: GCAGCAAACTGGAAAGGAAG
	GSDMD	F: GGTTCTGGAAACCCCGTTAT
		R: CCAGGTGTTAGGGTCCACAC
	NF-κB1	F: CCTGGATGACTCTTGGGAAA
		R: TCAGCCAGCTGTTTCATGTC
	GAPDH	F: ACAGTCAGCCGCATCTTCTT
		R: GACAAGCTTCCCGTTCTCAG

TRPV4, transient receptor potential vanilloid 4; TJP-1, tight junction protein 1; CYBA, cytochrome B-245 alpha chain; CASP1, caspase-1; ASC, apoptosis-associated speck-like protein containing a caspase recruitment domain; NLRP3, nucleotide-binding oligomerization domain, leucine rich repeat and pyrin domain containing 3; GSDMD, gasdermin D; NCF-1, neutrophil cytosolic factor 1; F, forward; R, reverse.

**Table II:** Baseline characteristics and biochemical indicators of serum and urine samples.

Characteristic	Control group	High salt diet group	P-value
Weight at week 8, g	27.35±0.75	23.48±1.33	<0.001
Water consumption, ml/week	35.54±4.60	82.97±7.92	<0.001
Serum biochemistry, mmol/l			
Na^+^	149.40±1.50	146.80±2.04	0.074
C^l^-	103.68±0.95	107.25±14.36	0.592
Urinary biochemistry			
Na^+^, mmol/l	75.20±40.29	396.67±98.49	<0.001
Cl^-^, mmol/l	87.08±39.22	455.17±146.12	<0.001
K^+^, mmol/l	166.18±19.03	121.07±50.55	0.122
Ca^2+^, mmol/l	0.83±0.21	0.84±0.40	0.962
Urine protein, µg/l	47.07±3.47	73.61±9.68	<0.001

Data are presented as mean ± SD (n=6). P-values were calculated using a two-tailed unpaired Student's t-test.

## Data Availability

The data generated in the present study may be requested from the corresponding author.
